# Validation of the Chinese version of the physical education teacher job satisfaction scale

**DOI:** 10.3389/fpsyg.2022.1040388

**Published:** 2022-11-29

**Authors:** Junfeng Yuan, Liping Zhang, Shaojing Weng, Yujia Yin, Chen Li, Lin Luo

**Affiliations:** ^1^School of Physical Education, Guizhou Normal University, Guiyang, China; ^2^School of Physical Education, Guiyang College, Guiyang, China; ^3^Basic Education Research Center, Southwest University, Chongqing, China

**Keywords:** physical education teachers, job satisfaction, self-efficacy, PETJSS, validation

## Abstract

**Purpose:**

The purpose of this study was to test the factor structure as well as the reliability of the Physical Education Teacher Job Satisfaction Scale (PETJSS).

**Method:**

The scale’s structural validity, internal consistency and reliability were examined using CFA and Cronbach alpha. The predictive validity of the PETJSS was examined using Teacher Self-Efficacy (TSES-11) and the personal characteristics of the subjects.

**Result:**

The three-factor structure of the PETJSS was confirmed. The PETJSS three-dimensional model had good internal consistency/reliability. The three dimensions of the PETJSS (colleague satisfaction, parent satisfaction and student behaviour satisfaction) explained 81.206% of the overall job satisfaction. Also, the PETJSS demonstrated the expected correlation with teachers’ self-efficacy, whilst the PETJSS test results were related to physical education teachers’ job titles.

**Conclusion:**

The PETJSS (Chinese version) can be considered as a valid and reliable method.

## Introduction

Although job satisfaction is used in scientific research and in everyday life, there is no universally accepted definition of job satisfaction in academia (Ghazzawi, 2008). Researchers from different disciplines have different theoretical approaches and frameworks for the study of job satisfaction. For example, in the field of psychology, job satisfaction was defined as employees’ emotional responses to their work environment (Sypniewska, 2014; Yousef, 2016). Pandey and Asthana (2017) defined optimism based on employees’ desired outcomes as job satisfaction, a view that considers job satisfaction as the positive impact of job-related experiences on an individual’s (Toropova et al., 2021). In sociology, on the other hand, it was seen as a different category of variable related to how each employee evaluates and thinks about his job (Taheri et al., 2020). Job satisfaction was viewed as a result of employees’ interactions and perceptions with their workplace and surroundings (Asrar-ul-Haq et al., 2017; Pongton and Suntrayuth, 2019). Although studies have approached the phenomenon of job satisfaction in different ways, researchers agree that job satisfaction is a multidimensional concept that consists of many components (Munir and Rahman, 2016; Sinha et al., 2022).

In the literature, teacher job satisfaction was found to be positively associated with teacher turnover (Hee et al., 2019), teaching attitudes (Cunningham, 2016), positive relationships with students (Banerjee et al., 2017), and with teacher anxiety, depression (Capone and Petrillo, 2020), and job stress (Troesch and Bauer, 2017) were negatively correlated. “Satisfied” teachers contributed to improved organisational performance and demonstrated high levels of job commitment (McCarthy et al., 2014). “Satisfied” teachers had higher self-efficacy, which in turn influenced students’ academic performance (Tsai and Antoniou, 2021).

In teacher professional psychology, it is crucial to have a reliable instrument to measure teachers’ job satisfaction (Sahito and Vaisanen, 2020). This would contribute to enhancing the management and services provided to teachers in schools, reduce teacher occupational stress (Nagar, 2012) and burnout (Skaalvik and Skaalvik, 2017), and promote teacher job satisfaction (Akomolafe and Ogunmakin, 2014). It is therefore important to develop easy-to-apply tools for school administrators and researchers to measure teacher job satisfaction.

In order to assess teacher satisfaction quantitatively, researchers have developed a number of operationalised instruments for assessing teacher job satisfaction. Scarpello and Campbell (1983) advocated the use of single-item measures to assess job satisfaction, i.e., “How satisfied are you with your job?” They argued that individual items take up less time, were more cost-effective, and could be used to monitor satisfaction on a daily basis. However, in dynamic and complex settings, researchers rarely use single-item instruments to measure teacher job satisfaction, and most questionnaires are multidimensional or multiple. For example, Lester (1987) developed the Teacher Job Satisfaction Questionnaire (TJSQ) containing nine dimensions, which are supervision, colleagues, working conditions, pay, responsibility, work (itself), advancement, security, and recognition. Hirschfeld (2000) assessed 20 aspects of teachers’ job satisfaction through 100 items. Ho and Au (2006) developed the Teaching satisfaction scale (TSS), a five-item questionnaire that asks teachers about their perceptions of job satisfaction in a variety of ways. Pepe (2011) developed the Teacher Job Satisfaction Scale (TJSS-9), a three-dimensional, nine-item scale that includes colleague satisfaction, parent satisfaction and student behaviour satisfaction. Although research on teacher job satisfaction has been conducted for decades, scholars are also increasingly looking at the quality of teacher job satisfaction assessment instruments and their applicability to teachers of different disciplines (Chalghaf et al., 2019).

A review of the literature revealed that few studies had investigated Chinese primary and secondary school physical education teachers’ perceptions of their job satisfaction. To our knowledge, there was no Chinese version of a psychological measurement instrument to assess the job satisfaction of Chinese primary and secondary school PE teachers. Whereas primary and secondary school physical education teachers are the main implementers of school physical activity and health promotion for students at the basic education level, the job satisfaction of primary and secondary school physical education teachers is a topic of concern in the Chinese educational environment.

Therefore, this study aimed to find a reliable teacher job satisfaction measure to assess the job satisfaction of Chinese primary and secondary school physical education teachers. The TJSS-9 developed by Pepe (2011) has been cross-culturally adapted and validated with physical education teachers in Arabic-speaking countries and has obtained good internal consistent reliability/confidence, predictive validity and sensitivity validation results. Pepe’s three-dimensional theoretical model of the TJSS-9 considered the teacher-student relationship, clearly the first dimension of teacher job satisfaction, on which there is now consensus amongst researchers (Spilt et al., 2011; Addimando, 2013). A common source of job stress for teachers is their interaction with students, classroom management difficulties, which is a key factor in stress and burnout later in a teacher’s career (Veldman et al., 2013). The second dimension of the TJSS-9 is also related to the social climate in the work organisation, mainly the impact of collegiality on individual job satisfaction. Luthans (2002) suggested that this factor as the main determinant of job satisfaction. Finally, in line with current thinking on the social aspects of teachers’ work, the third dimension included in the model is satisfaction with parents. Extensive research has explored the importance of parental involvement on children’s academic performance, suggesting that families should be fully involved in the school process (Fan and Chen, 2001; Jeynes, 2010). The TJSS-9 has achieved good measurement invariance in international cohorts from Netherlands, Russia, Hong Kong, China, the United States, Italy and Palestine. Chalghaf et al. (2019) applied the TJSS-9 to physical education teachers in Arabic departments and achieved good measurement invariance. The TJSS-9 has previously been validated well in a Hong Kong, China sample. However, as there are many differences between the education systems and management models in Mainland China and Hong Kong, the applicability of the TJSS-9 to the assessment of job satisfaction of physical education teachers in primary and secondary schools in China needs to be tested with an empirical sample. Therefore, the main objective of this study was to validate the psychometric properties of the Chinese version of the Physical Education Teacher Job Satisfaction Scale (PETJSS) on the basis of the three-dimensional theoretical model of the TJSS-9. The main objective of this study was to validate the psychometric properties of the Chinese version of the PETJSS and to determine the factor structure of the PETJSS and its measurement invariance in a sample of Chinese primary and secondary school physical education teachers.

## Materials and methods

### Participant

The sample consisted of 764 physical education teachers from primary and secondary schools in China. 64.92% were male and 35.08% were female. 49.74% were primary school physical education teachers, 30.37% were middle school physical education teachers, 15.71% were high school physical education teachers and 4.19% were physical education teachers from other educational institutions. Age: 46.07% were under 30 years old, 31.94% were 31–40 years old, 19.37% were 41–50 years old and 2.62% were 51–60 years old. Education level: high school/high school/secondary school and below 0.52%, college and bachelor’s degree 94.24%, master’s degree and above 5.24%. 33.51% in rural, 66.49% in urban. Years of teaching experience: 44.50% for <5 years, 24.61% for 6–10 years, 10.47% for 11–15 years, 3.14% for 16–20 years, 13.09% for 21–25 years, 3.14% for 26–30 years, and 1.05% for 30 years and above. All subjects signed an informed consent form and volunteered to participate in the survey. Questionnaires were administered electronically to all participants. Questionnaires were completed anonymously. The sample was collected from July 3, 2022 to October 26, 2022.

Ethical approval for the research protocol of this survey was obtained from the Academic Committee of the School of Physical Education, Guizhou Normal University (No. 20220630). An electronic informed consent form was set up on the first page of the questionnaire for this study. Teachers were made widely aware of the purpose and procedures of the study and were informed that the results would be made available to them upon completion of the study in summary form only, with no possibility of tracing individual teacher scores, thus ensuring anonymity and protecting the privacy of each participant. The survey was conducted in accordance with the ethical principles of the 1964 Declaration of Helsinki and its subsequent amendments.

### Instrument

The Teacher Job Satisfaction Scale (TJSS-9; Pepe, 2011) is a questionnaire designed to measure teacher job satisfaction and was developed specifically for use in educational settings. The TJSS-9 consists of nine items in three dimensions. The three dimensions are colleague satisfaction (three items), parent satisfaction (three items) and student behaviour satisfaction (three items). The items are coded using a five-point Likert scale for response making. The original version of the TJSS-9 was written in English. The TJSS-9 is a modified and simplified version of the initial six dimensions of 35 items. The TJSS-9 has a more robust, reliable and compact measurement model.

The Chinese version of PETJSS was completed in three steps. Firstly, two authors (W.S.J and Z.L.P) translated the English version of the TJSS-9 into Chinese and referred to the study by Chalghaf et al. (2019). Adding the definition of the environment of physical education work to the description of the work environment. Secondly, the linguistic expressions were discussed and revised collectively by two linguistics professors. Third, a pre-reading group of 10 physical education teachers was recruited to pre-reading the Chinese version of the PETJSS in order to revise the way the language was described that was deemed inappropriate. The PETJSS has a total of nine items, one dimension for every three items. The answers to the PETJSS items were coded using a five-point Likert scale. The English and Chinese descriptions of the Chinese version of the PETJSS are shown in Table 1.

**Table 1:** English and Chinese versions of PETJSS.

Code	Item	项目
A1	The quality of your relationships with your colleagues of sports and physical education at work	您在体育教育工作中与同事的关系?
A2	The extent to which your colleagues of sports and physical education encourage and support you in your work	您在体育教育工作中获得同事鼓励和支持的程度?
A3	Your overall satisfaction with your colleagues of sports and physical education	你对体育教育工作中同事的满意程度?
A4	The extent of students’ self-discipline behaviour in the sports and physical education class	您的体育课上学生自律吗?
A5	Your satisfaction with the behaviour of students in the sports and physical education class	您对体育课上学生行为的满意程度
A6	The overall level of satisfaction with students’ discipline in sports and physical education class	您对体育课上学生体育成绩的满意程度?
A7	The degree of interest shown by parents towards their children being taught sports and physical education	您的学生家长对孩子学习体育的兴趣程度?
A8	The extent to which parents support the school and its programs in sports and physical education	您的学生家长对学校体育教育的支持程度?
A9	Your overall level of satisfaction with parents where you work	您对学生家长的总体满意程度?

The Teacher Self-Efficacy Questionnaire (TSES-11), was designed to assess teachers’ self-efficacy in educational work settings (Kalkan, 2020). It was used as a means of cross-validating PETJSS scores in this study for the following main reasons: (a) Teacher self-efficacy is again a high predictor of teacher job satisfaction (Caprara et al., 2006; Kalkan, 2020). (b) The Chinese version of the questionnaire has been well used in China (Ma et al., 2019), with satisfactory results in terms of score reliability and normality of the distribution. The Cronbach alpha values and confidence intervals for the TSES-11 questionnaire scores were: *α* = 0.801, 95% CI [0.783, 0.809].

### Statistical analysis

Stata17 and AMOS 23.0 software were used for statistical analysis. Descriptive statistics were used to analyse the demographic characteristics of the sample, such as frequencies and percentages for categorical variables, and means and standard deviations for continuous-type variables. Assumptions related to factor analysis (e.g., normality, etc.) were checked for all variables between analyses to avoid cases of overly skewed distributions. Outliers were identified by *p* < 0.01. As there is no single statistical significance test to determine the correct model for a given data sample, the study recommended that the test consider the goodness of fit of multiple indicators (Lance et al., 2016). In line with this recommendation, the indicators of model fit chosen for this study were the Comparative Fit Index (CFI), Tucker-Lewis Index (TLI), Root Mean Square Error of Approximation (RMSEA) and Standardised Root Mean Square Residual (SRMR) to test (Bentler and Bonett, 1980). To obtain evidence of discriminant validity for the factors that comprise the instrument, this study used validated factor analysis CFA (estimation method: maximum likelihood) to assess three different models for the entire sample. The first (M1), was to build a robust baseline PETJSS model for further analysis. M1 loaded all items onto a single one-dimensional factor. Then, the fit of the two-dimensional model (M2) and the three-dimensional model (M3) was continued to be evaluated to compare the fit strengths and weaknesses of the different models through the fit metrics. To avoid the possibility of overfitting, we applied exploratory structural equation modelling ESEM for a mixed approach of EFA and CFA to assess the factorial validity of the selected optimal models (Satorra and Bentler, 2001; Li, 2016). The results of CFA and ESEM were interpreted according to the following commonly used model fit cut-off criteria: *χ*^2^/df ≤ 3, CFI > 0.90, TLI > 0.90, RMSEA <0.10 and SRMR <0.08. A good criterion for CFA and ESEM is that each latent variable factor should be >0.5, ideally >0.7 (Hair, 2009). For discriminant validity, a correlation coefficient of <0.85 between both factors was used as a criterion for validity (Kline, 2015). The internal consistency of the scale was tested using Cronbach’s *α* coefficient, which was >0.7 (Viladrich et al., 2017). Scale items were tested for measurement invariance based on published guidelines for building model measurement invariance (Pepe et al., 2017). After determining the validity and reliability of the PETJSS, TSES-11 scores were used to analyse its correlation with job satisfaction scores. Statistically significant correlations between PETJSS scores and TSES-11 scores imply concurrent validity.

## Results

### Preliminary analysis

Preliminary analysis showed that none of the items had missing, discrete or invalid values. Table 2 shows item correlations, means, standard deviations, skewness and kurtosis. The correlation matrix for all items showed that all items had statistically significant correlations (*p* < 0.01). The mean PETJSS total score for the nine items was 33.587 (SD = 5.525). The skewness and kurtosis results for the nine items of the PETJSS (Table 3) suggest that the normality assumption is invalid (Kline, 2015). Therefore, we believe that the maximum likelihood estimator (MLR) is appropriate for the CFA and ESEM (Tabachnick et al., 2007) calculations.

**Table 2:** Pearson correlation of PETJSS items.

Code	Mean	SD	A1	A2	A3	A4	A5	A6	A7	A8
A1	4.119	0.886								
A2	3.913	0.886	0.695**							
A3	3.956	0.747	0.607**	0.755**						
A4	3.438	0.758	0.325**	0.507**	0.545**					
A5	3.644	0.721	0.431**	0.620**	0.636**	0.701**				
A6	3.594	0.746	0.368**	0.469**	0.600**	0.572**	0.687**			
A7	3.656	0.832	0.226**	0.377**	0.441**	0.529**	0.644**	0.564**		
A8	3.663	0.784	0.330**	0.464**	0.544**	0.546**	0.654**	0.624**	0.785**	
A9	3.606	0.778	0.333**	0.488**	0.609**	0.561**	0.645**	0.644**	0.664**	0.781**

**p < 0.01.

**Table 3:** Normality test results for PETJSS items.

Code	Mean	SD	Skewness	Kurtosis	Kolmogorov–Smirnov Test	
					*D*-value	Value of *p*
A1	4.119	0.886	−1.390	2.890	0.278	<0.001
A2	3.913	0.886	−0.487	−0.452	0.252	<0.001
A3	3.956	0.747	−0.204	−0.504	0.261	<0.001
A4	3.438	0.758	0.302	−0.229	0.293	<0.001
A5	3.644	0.721	0.248	−0.491	0.264	<0.001
A6	3.594	0.746	0.271	−0.456	0.274	<0.001
A7	3.656	0.832	0.056	−0.338	0.254	<0.001
A8	3.663	0.784	0.121	−0.165	0.263	<0.001
A9	3.606	0.778	0.087	−0.029	0.263	<0.001

### Internal consistency

Table 4 lists the key indicators of internal consistency for the PETJSS. Corrected item total correlations (CITC) ranged from 0.520 to 0.813, indicating that all nine items were suitable for scale construction. The Cronbach alpha coefficient for the PETJSS was 0.915, indicating that the scale is reliable (Tabachnick et al., 2007). The alpha coefficients for the items that have been removed are all above 0.80, indicating that the data are of high reliability quality and can be used for further analysis. The results of the other Cronbach alpha coefficient analyses are also presented in Table 4. The results indicated that removing an item had no significant effect on the Cronbach alpha coefficient.

**Table 4:** Corrected item correlation statistics for PETJSS items.

Code	Correction item total correlation (CITC)	Item deleted alpha coefficient	Cronbach *α*
A1	0.520	0.920	0.915
A2	0.702	0.906	
A3	0.769	0.901	
A4	0.679	0.907	
A5	0.813	0.899	
A6	0.721	0.905	
A7	0.665	0.908	
A8	0.758	0.902	
A9	0.757	0.902	

### Factor validity

Use KMO to check for bias correlation between variables. The closer the KMO value is to 1, the stronger the biassed correlation between the variables and the better the factor analysis will be. The KMO of the questionnaire was 0.892, indicating a strong bias correlation between variables. The Bartlett’s sphericity test was used to determine whether the correlation matrix was a unitary array.

**Figure 1:**
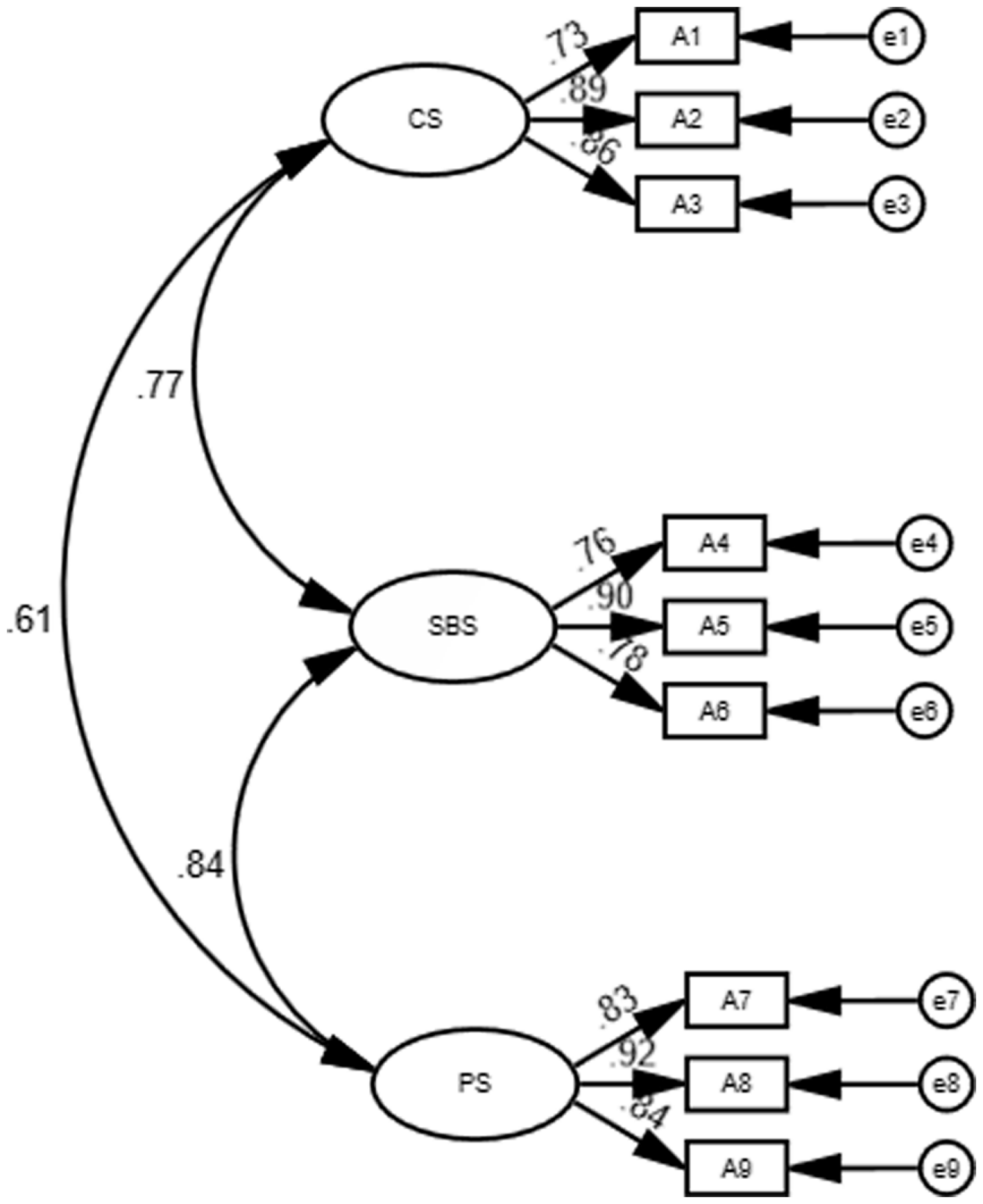
PETJSSI three-dimensional model. CS, Colleague Satisfaction; SBS, Student Behaviour Satisfaction; PS, Parent Satisfaction.

The data passed the Bartlett’s sphericity test (*p* < 0.05). The results of the KMO and Bartlett’s sphericity tests indicated that the questionnaire was suitable for further factorial validation. The CFA results for the initial measurement model (M1) reported poor factor validity. The one-dimensional structure of the PETJSS, whilst meeting the criterion of all factor loadings being >0.4, failed to meet most of the criteria for a good model. A two-dimensional model was then fitted to the PETJSS on its basis (M2). In M2, A1–A3 were classified as one dimension and A4–A9 as the other in terms of factor loadings. The fit metrics for M2 showed a decrease in *χ*^2^/df, an increase in CFI and TLI, and a decrease inRMSEA and SRMR. Although the fit metrics for M2 improved to some extent, they still fell short of the recommended range and the factor loadings for each of the items in M2 were above 0.4.The fitting of the three-dimensional model (M3) was then continued on the basis of M2 with factor loadings based on the three-dimensional divisions. In M3, A1–A3 were classified as one dimension, A4–A6 as one dimension and A7–A9 as one dimension in terms of factor loadings. Compared to M2, the fit indices for M3 showed a decrease in *χ*^2^/df, reaching the criterion of *χ*^2^/df < 3. CFI and TLI increased, reaching the criterion of CFI, TLI > 0.9. RMSEA and SRMR decreased, reaching the criterion of RMSEA <0.1 and SRMR <0.08. M3 showed a satisfactory fit index, indicating that it should be accepted. The three dimensions of M3 (colleague satisfaction, parent satisfaction and student behaviour satisfaction) were consistent with the three dimensional divisions of the TJSS-9, explaining 81.206% of the overall job satisfaction of primary and secondary PE teachers. The fit indices of the PETJSS model are shown in Table 5. The relationships between the items and satisfaction dimensions of M3 are reported in Figure 1.

**Table 5:** Fitting indicators for the PETJSS model.

	*χ*^2^/df	CFI	TLI	RMSEA	SRMR
M1	8.195	0.807	0.743	0.213	0.093
M2	4.243	0.916	0.884	0.143	0.066
M3	2.556	0.959	0.970	0.089	0.052

### Predictive validity

Table 6 presents the relationship between PETJSS scores and TSES-11 scores. Pearson correlation analysis revealed that TSES-11 scores were positively correlated (*p* < 0.01) with scores on all three dimensions of the PETJSS (colleague satisfaction, student behaviour satisfaction and parent satisfaction). These correlations are consistent with other previous studies exploring the relationship between job satisfaction and teacher self-efficacy (Caprara et al., 2006).

**Table 6:** Pearson correlation between PETJSS scores and TSES-11 score.

	PS	SBS	CS
TSES-11 score	0.290**	0.340**	0.270**

**p < 0.01.

Table 7 presents the results of the multiple linear regression of PETJSS scores with physical education teachers’ gender, age, occupation, residence, years of teaching experience, educational experience and job title. The results showed that PETJSS scores were positively correlated with teachers’ job titles only (*p* < 0.05).

**Table 7:** Multiple linear regression results of PETJSS scores and personal characteristics of physical education teachers.

	Non-standardised coefficient		Standardised coefficient	*t*	Value of *p*
	*B*	Standard error	Beta		
Gender	−1.014	0.957	−0.087	−1.060	0.291
Age	0.155	0.901	0.024	0.172	0.864
Occupation	0.787	0.598	0.115	1.318	0.190
Education level	1.800	1.850	0.080	0.973	0.332
Job title	2.763	1.197	0.219	2.309	0.022*
Years of teaching experience	−0.476	0.475	−0.143	−1.002	0.318
Residence	−0.106	0.935	−0.009	−0.113	0.910

*p < 0.05.

## Discussion

The purpose of this study was to validate the validity of the Teacher Job Satisfaction Scale (TJSS-9) amongst Chinese primary and secondary school physical education teachers. The PETJSS was translated from Pepe’s TJSS-9 three-dimensional model and referenced from Chalghaf et al. (2019) by adding physical education work to the description of the work environment in the definition of environment. The findings suggest that the three-dimensional structure of the PETJSS was validated in a sample of Chinese primary and secondary school physical education teachers. Both ESEM and CFA were used in this study. The ESEM factors loaded well and the CFA fit indices were satisfactory. After validation, the three-dimensional model was found to have good internal consistency/reliability. The three dimensions of the PETJSS (colleague satisfaction, parent satisfaction and student behaviour satisfaction) explained 81.206% of the overall job satisfaction of primary and secondary school physical education teachers.

To our knowledge, no study to date has used a sample of Chinese primary and secondary school physical education teachers to validate the adaptation of the TJSS-9 in a Mandarin Chinese context. This is despite the fact that the TJSS-9 has been previously validated for measurement invariance in six countries/regions (Netherlands, United States, Russia, Hong Kong, China, Italy and Palestine) with 2,819 teachers. The validation results showed that the TJSS-9 demonstrated strong psychometric properties, with no significant differences between groups in terms of measurement invariance (Pepe et al., 2017).

The results of this study showed that job satisfaction was only related to the job title of primary and secondary school physical education teachers. That is, job title was a significant independent predictor of job satisfaction amongst primary and secondary school physical education teachers in China. Sahito and Vaisanen’s (2020) study found that job title affects teacher satisfaction in developing countries. Tolliver’s (2018) study reported that job title helps to increase primary school teachers’ job satisfaction. Aytac’s (2020) study identified that job title significantly affects job satisfaction of teachers in both public and public schools. Some previous studies have found that there may also be gender differences in teachers’ job satisfaction. In Topchyan and Woehler’s (2021) study, female teachers had slightly higher job satisfaction than males. In addition, other scholars (Sak, 2018; Magee, 2013) suggested that gender may have a direct or indirect relationship with job satisfaction. However, the results of Oshagbemi’s (2000) study supported that gender does not affect teachers’ job satisfaction. The study by Lüleci and Çoruk (2018) reported that age did not affect teachers’ job satisfaction. This study also did not find a significant effect of age on job satisfaction of physical education teachers. Whilst Crisci et al.’s (2019) study reported that age affects teachers’ job satisfaction.

This study did not find that the occupation of the teacher had an effect on the job satisfaction of physical education teachers. In contrast, some previous studies found significant differences in the levels of job satisfaction amongst primary, secondary or high school teachers. For example, Demirtas (2010) reported that primary school teachers had higher job satisfaction than secondary school or university teachers. Buyukgoze-Kavas et al. (2014) reported higher job satisfaction amongst Turkish teachers in primary and secondary schools than amongst secondary school teachers. Indhumathi (2011) conducted a study amongst teachers in a secondary school and there were significant differences in job satisfaction amongst teachers depending on their grade level. In addition, some studies had found that teachers’ self-efficacy was a significant predictor of teachers’ job satisfaction. For example, Collie et al. (2012) reported that teachers’ job satisfaction was directly related to teaching self-efficacy. This was consistent with the findings of this study. From a methodological perspective, based on the experience of developing the Chinese version of the PETJSS in this study, it is possible to derive overall and specific dimensions of PE teachers’ job satisfaction, which will help in assessing and understanding the constructs studied. The short duration of the Chinese version of the PETJSS assessment, the low burden of questions and the ease of interpretation of the scores encourage that the PETJSS can be applied to different educational settings at different stages of basic education in China. The Chinese version of the PETJSS can therefore be categorised as a short and user-friendly measure of job satisfaction, designed to make data collection as easy as possible whilst avoiding overburdening individuals working in dynamic organisations (e.g., schools).

This study also had limitations that are worth discussing. Firstly, the research design is cross-sectional. Therefore, a further interesting development would be a longitudinal follow-up of the patterns of job satisfaction across different groups of teachers. Secondly, the sample size for this study was relatively small, although it met the sample size requirement of 5–7 times the scale question size. Thirdly, the sample size of rural teachers in our sample was small. Although we attempted to compensate for sampling error by increasing the sample size, the scope for generalising our findings to a broader group of teachers remains limited. A final limitation comes from the TJSS-9 itself, a measurement model that only includes satisfaction with social relationships (colleagues, parents and students) and does not include other factors that influence job satisfaction, such as organisational culture, work climate and pay. Therefore, it is also important to refine and add to the Chinese version of the PETJSS in the future in order to obtain a complete assessment of job satisfaction amongst physical education teachers.

## Conclusion

The Chinese version of the Physical Education Teacher Job Satisfaction Scale (PETJSS) is a measure of job satisfaction for physical education teachers. The scale is based on the TJSS-9 three-factor model, which analyses colleague satisfaction, parent satisfaction and student behaviour satisfaction. This study supports the sub-dimensional model of the PETJSS and demonstrates measurement invariance amongst Chinese primary and secondary school physical education teachers. In addition PETJSS demonstrated the expected correlation with the reference instrument. In conclusion, the Chinese version of the PETJSS is a valid and reliable measure.

## Data availability statement

The raw data supporting the conclusions of this article will be made available by the authors, without undue reservation.

## Ethics statement

The ethical review of this study was reviewed and approved by the Academic Committee of the School of Physical Education, Guizhou Normal University (No. 20220630). Patients/participants provided their written informed consent to participate in this study.

## Author contributions

JY and LL were responsible for design, data statistics and text writing. LZ and SW were responsible for literature search and scale translation. YY and CL were responsible for data collection. LL was responsible for final reading of the manuscript. All authors contributed to the article and approved the submitted version.

## Funding

The research was funded by the Guizhou Provincial Department of Education Youth Growth Project Fund (Qianjiao He KY (2021) 291), the Guizhou Province Education Planning Fund Project (2021A058) and the Guizhou Normal University Curriculum Thinking and Government Teaching Reform Project (2022(60).

## Conflict of interest

The authors declare that the research was conducted in the absence of any commercial or financial relationships that could be construed as a potential conflict of interest.

## Publisher’s note

All claims expressed in this article are solely those of the authors and do not necessarily represent those of their affiliated organizations, or those of the publisher, the editors and the reviewers. Any product that may be evaluated in this article, or claim that may be made by its manufacturer, is not guaranteed or endorsed by the publisher.
